# Browser-based design of loop-mediated isothermal amplification (LAMP) primer sets with template repeat masking and bisulphite support

**DOI:** 10.3389/fbinf.2026.1992214

**Published:** 2026-10-26

**Authors:** Ruslan Kalendar

**Affiliations:** 1 Helsinki Institute of Life Science (HiLIFE), University of Helsinki, Helsinki, Finland; 2 National Laboratory Astana, Nazarbayev University, Astana, Kazakhstan

**Keywords:** dimer screening, LAMP, linguistic complexity, loop-mediated isothermal amplification, primer design, primer dimers, repeat masking, web tool

## Abstract

Loop-mediated isothermal amplification (LAMP) requires four to six oligonucleotides to occupy six to eight defined regions of a target, with strict constraints on spacing and melting temperature. This makes manual design impractical; therefore, most users rely on a limited number of server-side design services. In this study, a browser-based LAMP primer set designer is presented that requires no installation or registration and does not transmit the target sequence. The tool generates six independent candidate lists per target and screens each candidate for melting temperature, linguistic complexity, simple sequence content, self-complementarity, and complementarity to existing user-owned oligonucleotides. The remaining candidates are then assembled into complete F3/F2/F1c-B1c/B2/B3 assays, optionally including loop primers. Any combination in which two members can form a stable duplex is first rejected by a fast combinatorial rule and then by the nearest-neighbor free energy of the most stable dimer that the assembled molecules can form at the reaction temperature. Regions of the template covered by dispersed repeats or low-complexity sequences are masked prior to design. The tool’s yield, parameter sensitivity, and masking behavior are reported, and its output is compared with an archived PrimerExplorer V5 design on an identical target. Four four-primer LAMP assays for transgene screening, which were designed using an earlier version of the same principles and validated experimentally, are also described. The tool is freely available at https://primerdigital.com/tools/lamp.html.

## 1 Introduction

Loop-mediated isothermal amplification (LAMP) can amplify a target to detectable levels in less than an hour at a single temperature using a strand-displacing polymerase, without the need for a thermal cycler (Notomi et al., 2000). The selectivity of LAMP comes from its primers rather than thermal cycling: the four core oligonucleotides recognize six separate regions of the target, and the two optional loop primers introduced by Nagamine et al. (2002) increase this number to eight while roughly halving the reaction time (Tomita et al., 2008). This redundancy makes LAMP attractive for field and point-of-care work, but it also makes primer design the most challenging aspect of developing an assay. The six oligonucleotides must be arranged in a specific order along approximately 200 bp of template, and they must form two distinct melting-temperature windows. Furthermore, none of them can pair with any other because a LAMP reaction is a single isothermal mixture in which all primers are present simultaneously, and no annealing step discriminates against mispriming events. Mispriming among the primers themselves is a documented source of false positives in LAMP and can be traced to individual members of a set (Hardinge and Murray, 2019); therefore, it is crucial to prevent this in the primer set. Design software for LAMP is therefore highly specialized. A recent survey of the field (Ptitsyn et al., 2024) found that most published assays were designed using PrimerExplorer (Eiken Chemical) or the New England Biolabs (NEB) LAMP Primer Design Tool. The remaining programs, LAVA (Torres et al., 2011), GLAPD (Jia et al., 2019), MorphoCatcher (Shirshikov et al., 2019), and LAMPrimers iQ (Akhmetzianova et al., 2024), are comparatively rare. These tools are capable and well established. The two most widely used are hosted services, whereby the target sequence is uploaded and the design is computed on a third-party machine. For clinical, agricultural biosecurity, or unpublished genome targets, this presents a real constraint. LAVA, MorphoCatcher, and LAMPrimers iQ are downloadable programs, but each requires a local Perl, C/CUDA, or Python toolchain, which introduces a different barrier; GLAPD is offered both as downloadable code and as a web server. Client-side design is no longer unique to the tool described here. Two further client-side implementations are now available. The ConductScience LAMP Primer Designer (ConductScience, 2026) is a free calculator page, published in April 2026 by a laboratory-equipment vendor and not described in a peer-reviewed article, which states that all computation runs client-side and that it designs F3, B3, FIP, BIP, and loop primers: it scans anchor positions along the target, selects the remaining components by nearest-neighbor melting temperature, and ranks the resulting sets on melting-temperature balance, GC content, and the Notomi spacing constraints (Notomi et al., 2000). Its documentation describes no screening for dimers between the primers of a set. primd (Handley, 2026) is an MIT-licensed TypeScript library for the browser and Node.js, whose LAMP module designs the same six components, with loop primers included by default, and screens candidates on hairpin, self-dimer, and hetero-dimer free energies at a settable annealing temperature; it is a library rather than a page, so it is used by writing code against it, and its documentation describes no masking of the template and no complexity filter. What distinguishes the present tool is therefore no longer that it runs in the browser. It is the combination of what happens before and after the geometry is satisfied: the template is masked for dispersed repeats and low-complexity sequence before any candidate is enumerated and every candidate is scored for linguistic complexity; candidates can be screened against the oligonucleotides a laboratory already holds; many records can be submitted at once; a bisulphite mode is provided; and the thermodynamic screen is applied after assembly, to the assembled inner primers as they would be synthesized, at the reaction temperature and at the ionic strength of a LAMP mixture rather than under a standard annealing condition. The tool described here is one of the design modules of a web platform for PCR and sequence analysis, which has previously been reported as a whole (Kalendar, 2025). This article specifically documents the LAMP module: what the design algorithm does, how its parameters behave, its performance on real targets, and its limitations. Three properties distinguish it from established tools. First, all computation is client-side JavaScript, meaning that the target never leaves the browser. Second, the target is masked for dispersed repeats and low-complexity sequences before candidates are enumerated. Every candidate oligonucleotide is then scored for linguistic complexity. The design refuses to place a primer on a sequence that is repeated elsewhere in the same template or is compositionally degenerate. This is precisely the type of sequence that gives rise to primer-derived background. Third, to ensure compatibility when designing multiplexing panels, users can apply previously used oligonucleotides. Any candidate that can pair with one of these is discarded, enabling a new assay to be designed that is compatible with existing sets.

## 2 Materials and methods

The design logic is contained within nine JavaScript modules (lamp.js, dna.js, dimers.js, dimers4.js, DNAtm.js, tsdr-nn.js, LowComplexitySequence.js, readseq.js, and tools.js), which are executed in the browser. The page also loads Bootstrap for the layout; however, this does not participate in the computation. There is no server component or build step. The design entry point (analysis() in lamp.js) processes each input record independently in three stages: template preparation and masking, enumeration of six candidate lists, and assembly of candidates into complete assays. The user-settable parameters and their defaults are listed in Table 1.

**TABLE 1:** User-settable design parameters, their defaults, and their effect. Parameter names are paraphrased; the corresponding on-page labels differ slightly in wording.

Parameter	Default	Range/values	Effect
Primer length	18–23 nt	Minimum 12–100 nt, maximum 12–500 nt	Seed length for the scan and the maximum 5′ extension
Tm range	60 °C–62 °C	Lower bound 37 °C–80 °C; upper bound unrestricted	Window for F3/F2/B2/B3; F1c, B1c, LF, and LB use this window +5 °C
Minimum linguistic complexity	70%	10%–90%	Rejects compositionally degenerate candidates
3′-end pattern	sws ssw sww wss www	Any IUPAC pattern list; n disables	Admits 40 of 64 3′ trinucleotides
Max F2-B2 amplicon	200 bp	100–350 bp suggested by the input field; the code enforces the upper bound only and no minimum	Upper bound only; no minimum is enforced
Max dimer ΔG at 65 °C	−6 kcal/mol	Any value; blank switches the screen off	Rejects assays whose most stable dimer among F3, B3, FIP, BIP, LF, and LB is below the limit; the value and the pair are reported for every assay
Non-specific priming control	On	On/off	Masks positions covered by repeated 15-mers
Low-complexity priming control	On	On/off	Masks low-complexity template blocks
Allow overlapping positions	Off	On/off	Increases the candidate overlap allowance from 12 to 18 nt
C→T bisulphite conversion	Off	On/off	Converts both templates; presets 17–28 nt, 57 °C–59 °C, 60% LC
Loop primer design	On	On/off	Adds LF and LB, giving six-primer, eight-region assays
My primers	Empty	Any oligonucleotide list	Discards candidates that could pair with existing stock

### 2.1 Input, targeting, and exclusion markup

Input can be pasted or uploaded as a file or retrieved by accession from NCBI. The accession method is the only process that contacts an external server and transmits the accession number, not the sequence. Plain sequence, single-record and multi-record FASTA, and name-sequence lines are all accepted, with no hard limit on record count or length. The parser deliberately avoids splitting on whitespace, meaning that position-numbered sequences pasted from a sequence viewer remain intact. Characters outside the IUPAC nucleotide alphabet were discarded: uridine (u) was read as thymine (t) and inosine (i) as guanine (g). Two markup characters control the location of the design. A region in square brackets restricts the search. The first bracketed region defines where the forward half-set (F3, F2, and F1c, as well as the forward loop primer) can be placed. The second bracketed region defines where the backward half-set can be placed. This allows the amplicon to be forced across a specific feature. With no brackets, both half-sets are searched over the entire record. Text between a pair of slashes is excluded from priming, which is the manual equivalent of the automatic masking described in the following subsection.

### 2.2 Template masking

Two masks were created from the template and combined additively with any user exclusion. The repeat mask is a single-record version of the TotalRepeats masking (Kalendar and Kairov, 2024; Kalendar, 2026) and counts every 15-mer on both strands of the record, marking each position covered by a 15-mer that occurs more than once. This is a self-comparison of the record against itself; it is not a search against a genome or a repeat database, nor is it the genome-wide TotalRepeat tool (Kalendar, 2026). It detects dispersed and tandem repetitions in the submitted sequence. These repetitions govern whether a primer designed for that sequence has more than one binding site. The low-complexity mask (LowComplexitySequence) slides an 18-nt window over the template and counts distinct dinucleotides and trinucleotides within it on a five-symbol alphabet, where all ambiguous codes are collapsed into one symbol. A window with a score below 14 out of a possible 33 initiates a block, which extends across up to 17 tolerated high-complexity positions and is retained if it spans more than 21 nucleotides (nt). Both masks were enabled by default. They act only where a record repeats itself, and on most templates, that means not at all: of the nine targets in Table 2, seven carry no repeated 15-mer, while a purpose-built tandem-repeat control is 90.4% masked, and the archived PrimerExplorer target, whose submitted 419 bp contains a genuine internal duplication, is 9.1% masked (Figure 1D). Masking also dominates the run time because it removes candidates before the combinatorial assembly stage rather than after.

**TABLE 2:** Yield at default settings across nine targets. Candidate counts are given per list. Masked fraction is the union of the repeat and low-complexity masks. Run times are medians of seven runs on one core, with the observed minimum and maximum in parentheses.

Target	Length (bp)	GC (%)	F	F1	LF	R	B1	LB	Candidates	Assays	Time (ms), median (min-max)	Masked (%)
Bundled example	391	48.6	23	15	16	26	16	15	111	5	102 (100–112)	0.0
PrimerExplorer V5 target	419	39.4	13	1	3	11	3	1	32	0	90 (89–95)	9.1
*NOS* promoter	291	46.7	12	10	9	14	9	10	64	0	70 (67–75)	0.0
*nptII* (neomycin)	804	59.7	37	46	43	36	43	46	251	19	155 (152–160)	0.0
*nptII* (kanamycin)	798	59.6	36	47	43	36	43	47	252	22	152 (145–153)	0.0
*bla* (ampicillin)	861	49.4	46	34	34	42	34	34	224	2	190 (182–196)	0.0
*P-FMV* promoter	554	41.2	18	11	13	22	13	11	88	0	131 (118–152)	7.4
*hpt* (hygromycin)	1,026	58.2	52	54	56	51	56	54	323	167	245 (226–251)	0.0
Tandem-repeat control	1,066	50.3	5	3	5	3	5	3	24	0	23 (22–25)	90.4

**FIGURE 1:**
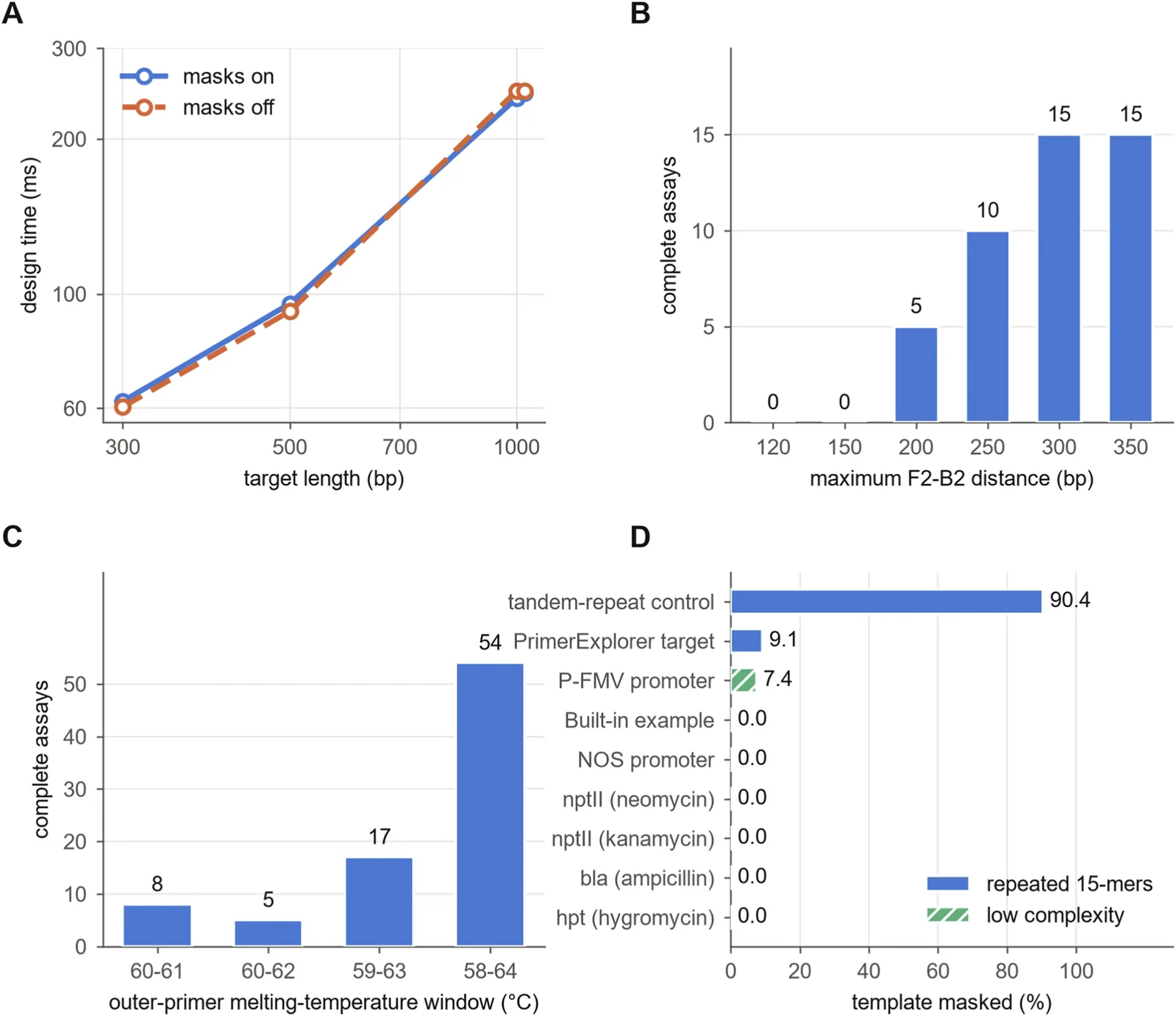
Performance and parameter behavior. **(A)** Design time against target length for truncations of the 1,026 bp *hpt* record, with template masking on and off; the record carries no repeated 15-mer, so the two series coincide and the design time tracks the candidate count. **(B)** Complete assays returned from the 391 bp example as a function of the maximum F2-B2 distance and **(C)** of the outer-primer melting-temperature window, all other settings at their defaults. **(D)** Masked fraction of each of the nine benchmark targets, separated into the repeat and low-complexity contributions.

### 2.3 Candidate generation

Six lists are enumerated independently, three per strand. On the plus strand, the tool builds the F list (which supplies both F3 and F2) and the F1 list; on the minus strand, it builds the R list (B3 and B2) and the B1 list. F1 and B1 are template regions: their reverse complements become the F1c and B1c segments of the inner primers. When loop-primer design is enabled (which is the default), a further list is built on each strand for the forward and backward loop primers (LF and LB) (the tool labels these lists FL and BL in its output; the conventional LAMP names are used here). The outer list is designed to the melting-temperature (Tm) window the user sets, and the inner and loop lists to that window plus 5 °C; with the default 60 °C–62 °C, this gives 65 °C–67 °C for F1c, B1c, LF, and LB, which is close to the 64 °C–66 °C that NEB recommends for loop primers. Enumeration is a left-to-right scan. At each position, the tool first requires that no base of the seed window is masked, then that the 3′-terminal trinucleotide matches the 3′-end patterns, and then grows the oligonucleotide 5′-ward from the minimum length until its melting temperature reaches the lower bound or the maximum length is exhausted. Only the seed window is mask-checked, so an accepted primer may overhang a masked block at its 5′ end by up to the difference between the maximum and minimum length (5 nt at the defaults). The default 3′-end pattern *sws ssw sww wss www* admits 40 of the 64 possible trinucleotides: the three classes not listed, all-S (*sss*), alternating *wsw* and *wws*, are excluded. It is a 3′-stability filter rather than a GC clamp, and it can be disabled by entering n. Surviving oligonucleotides must then pass, in the following order: the melting-temperature window; a simple-sequence blacklist (*gggg, ccccc, aaaaa, ttttt, gcgcgc, cgcgcg, atatat, tatata*); a linguistic-complexity threshold; a self-complementarity test; and a test against every oligonucleotide the user has pasted into the My Primers tab.

Linguistic complexity (LC) was computed on the candidate itself as the observed k-word vocabulary |V_k_| over the maximum possible vocabulary for an oligonucleotide of length l: LC % = ⌊100·Σ_k_|V_k_|/Σ_k_ min(l-k+1, 4^k^)⌋ for k = 1–3, following the linguistic-complexity measures of Gabrielian and Bolshoy (1999) and of Orlov and Potapov (2004). A second value (YR %) is reported over the purine/pyrimidine two-letter alphabet for k = 1–6; it is informative but is not used as a filter. The default threshold of 70% is strict: a perfect (ACGT)_5_ repeat scores 31% and a 20-mer of a single base scores 7%, but even a fairly ordinary-looking 20-mer can fall just under 70%.

The melting temperatures were determined using the unified nearest-neighbor parameters (Allawi and SantaLucia, 1997), extended with deoxyinosine (Watkins and SantaLucia, 2005), LNA (McTigue et al., 2004), and RNA (Xia et al., 1998) steps, and corrected for salt using the the monovalent and divalent relations of Owczarzy et al. (2004) and Owczarzy et al. (2008), selected on the ratio √[Mg^2+^]/[Mon], where [Mon] is the total monovalent cation concentration. Two salt conventions are used in this study, and it matters which quantity sits on which. Candidate melting temperatures are evaluated at one fixed condition set (55 mM monovalent cation, 1 mM Mg^2+^, and 0.2 µM oligonucleotide), shared by every design tool on the platform so that Tm values remain comparable between them; the melting temperature of the F2-B2 fragment and every dimer free energy reported here are evaluated instead at the 349 mM sodium equivalent of a LAMP mixture. That fixed condition set is a standard PCR profile, not LAMP chemistry, which typically runs at 6-8 mM Mg^2+^ and 1.6 uM inner primer; the tabulated candidate Tm should therefore be read as a relative design target, several degrees below what a LAMP-specific calculator would report for the same oligonucleotide. The melting temperature of the F2-B2 fragment is reported separately from an empirical GC-based relation evaluated at a sodium equivalent of 349 mM, computed as [Mon] + 120·√[Mg^2+^] with both concentrations in mM (55 mM monovalent, 6 mM Mg^2+^) following von Ahsen et al. (2001) (their dNTP-binding correction [Mon] + 120·√([Mg^2+^] − [dNTP]), is not applied), and therefore does not reflect LAMP-like ionic conditions. Finally, candidates that overlap a previously accepted candidate by more than 12 nt (18 nt when overlapping positions are permitted) are thinned, and the higher-complexity member is retained. This keeps the lists sufficiently short for the assembly stage to be tractable.

### 2.4 Assay assembly

Half-sets are enumerated per strand under the published LAMP geometry (Figure 2): the 5′ end of F2 lies at most 20 nt downstream of the 3′ end of F3 (0–19 intervening bases), and each loop-forming interval (F2 to the loop primer, and the loop primer to F1) spans approximately 0–40 nt, with the mirror-image constraints on the backward side; the four loop intervals are enumerated over 0–40 nt on the forward side and 0–39 nt on the backward side, the one-nucleotide difference following from the coordinate conventions used for the two strands rather than from any intended asymmetry. Every half-set is checked pairwise for duplex formation among its own members before it is retained, and the search is bounded at 11 half-sets per outer primer and approximately 5,000 half-sets per strand. The bounds are hard: when one is reached, enumeration stops for that outer primer or strand, and the remaining combinations are never formed; therefore, on candidate-rich targets, the caps silently truncate the design space, and the admissible totals reported below are themselves lower bounds. Assembly on a strand is attempted only when its outer list holds at least two candidates as F3 and F2 (or B3 and B2) are drawn from the same list. The forward and backward half-sets were then paired. A pair was admissible if F1 ended at or before the start of B1, if the F2-B2 distance did not exceed the user’s maximum, and if none of the 16 cross-strand oligonucleotide pairs (nine without loop primers) could form a stable duplex. The admissible pairs were then thinned greedily: a pair was reported only if its F2-B2 fragment was shorter than, or its summed linguistic complexity higher than, that of the pair reported immediately before it. The rule shortens the output but does not make the reported assays diverse, and it makes their number depend on the enumeration order rather than on the size of the design space; both points are returned to in Section 3.3. Duplex detection is a seed-and-extend complementarity search that indexes 5-mers, extends each seed allowing one mismatch, and counts an event when the extended match reaches six bases at a 3′ end or eight bases internally; it is a combinatorial rule, not a free-energy calculation. Assays were reported in the order in which they were found, with the F1c and B1c segments reverse-complemented and joined to F2 and B2 through a TTTTTT linker to give the forward and backward inner primers (FIP and BIP).

**FIGURE 2:**
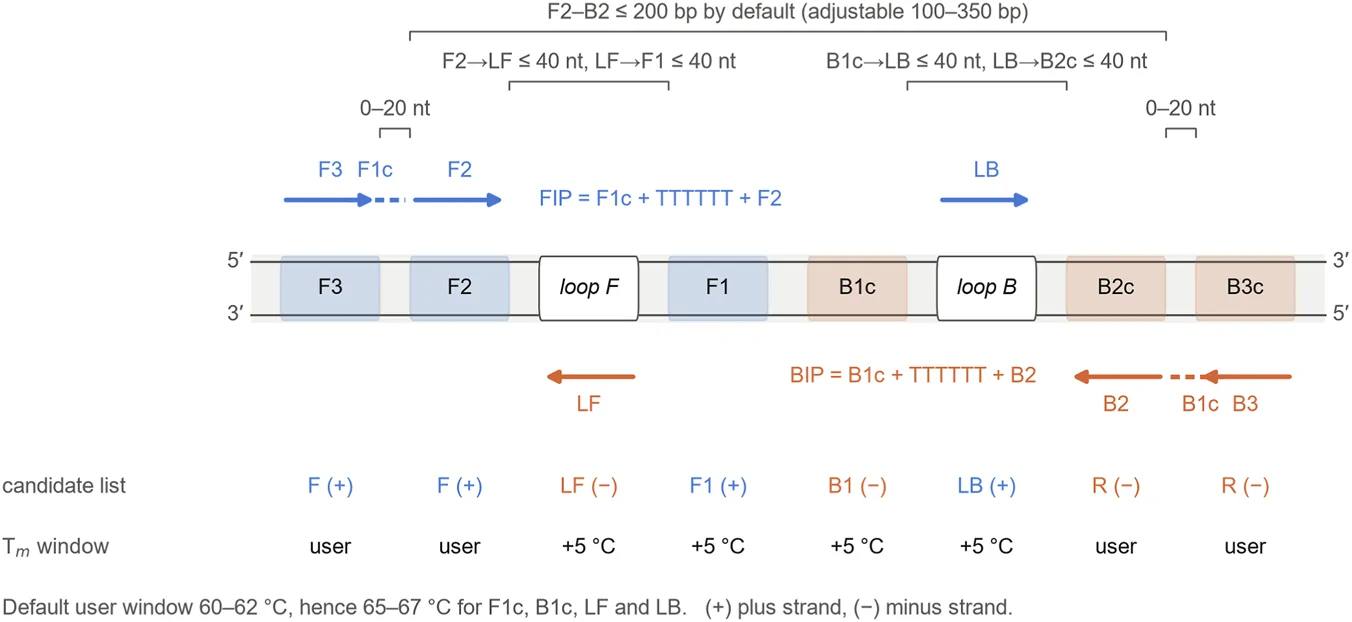
Geometry of a LAMP primer set and the candidate lists built for it. The six template regions are shown on the duplex, with the oligonucleotides read from each strand above and below it; F1c and B1c are the reverse complements of the F1 and B1 template regions and reach the tube only as the 5′ segments of FIP and BIP. Below the duplex, each region is labeled with the candidate list it is drawn from, the F list supplying both F3 and F2 and the R list supplying both B3 and B2, and with the melting-temperature window for which that list is designed. The spacing constraints enforced during assembly are marked above the duplex.

The combinatorial rule is a pre-filter. Once a forward and a backward half-set have been paired, the assay is screened a second time, thermodynamically, on the molecules that will actually be in the tube: F3, B3, the assembled FIP and BIP, and LF and LB when they are designed. Every pair and every self-pair among them are aligned by the seed-and-extend engine shared with the platform’s oligonucleotide-analysis tools (findDimersIndexed in dimers4.js), and the aligned core of each alignment, from the outermost Watson–Crick pair to the outermost Watson–Crick pair, is scored by a nearest-neighbor engine (TSDR_NN in tsdr-nn.js) that combines the unified Watson–Crick parameters (Allawi and SantaLucia, 1997; Santalucia, 1998; Santalucia and Hicks, 2004) with the measured parameters for internal single mismatches (Allawi and Santalucia, 1997; Peyret et al., 1999) and deoxyinosine (Watkins and Santalucia, 2005). The free energy is evaluated at the reaction temperature, 65 °C, with the entropy salt correction of Santalucia (Santalucia, 1998) at the same sodium equivalent of 349 mM used for the fragment Tm, so that the value describes a LAMP mixture rather than a standard-PCR one; it is therefore not comparable with dimer ΔG values quoted at 25 or 37 °C using other software. The most negative value over all pairs is the assay’s minimum dimer ΔG. Assays whose minimum lies below a user-set limit, −6 kcal/mol by default, are rejected, and the value and the pair responsible are reported for every assay that is kept; leaving the field blank switches the screen off. Results are memorized per pair of oligonucleotides because the same components recur in many assays, so the screen adds little to the run time.

### 2.5 Bisulphite mode

For methylation work, the tool converts the template before design in the conventional way for bisulphite primer design (Clark et al., 2006): the top strand is rewritten with every cytosine outside a CpG replaced by thymine, and the backward template is the reverse complement of that converted plus strand, so that the forward and backward half-sets are both designed against the same converted strand and can co-amplify it. Holding the CpG positions unconverted means that primers are designed against the fully methylated allele; the two converted strands of a bisulphite-treated duplex are no longer complementary, so an assay for the other strand requires a separate design on the reverse complement of the input. Enabling the mode relaxes the defaults to 17–28 nt, 57 °C–59 °C, 60% linguistic-complexity, and overlapping positions, which a cytosine-depleted, AT-rich converted template requires.

### 2.6 Output

Two tables were produced. The first lists every candidate that survived filtering, with its automatically generated positional name, sequence, length, Tm, GC content, and two linguistic-complexity values. The second lists the complete assays: F3, F2, LF, F1, the assembled FIP, B1, LB, B2, B3, and the assembled BIP, closed by the F2-B2 fragment size and its melting temperature. Both tables are tab-delimited and can be copied or downloaded directly as spreadsheet-ready files.

### 2.7 Benchmarking

All measurements below were obtained by loading the design modules into a Node.js v24.17.0 vm context with a minimal DOM stub and calling the same analysis() entry point the page calls, so that the design code under test is byte-identical to the code the browser runs. Timings are medians of seven runs for the measurements in Tables 2 and 3 and of three runs for the parameter sweeps reported in the Supplementary Material, on one core of an Intel Core i5-4570 at 3.20 GHz under Windows 10 (build 19045); they are therefore a measure of the algorithm rather than of any particular browser. As a spot check, the worked example was also timed in Chromium 148 on the same machine from the page itself: 54 ms at default settings (median of five runs) and 605 ms with overlapping positions allowed (median of three), against 103 ms and 788 ms under Node.js, so the browser figures are, if anything, lower. The harness, the nine target sequences, the design modules, and the complete output tables are provided as Supplementary Material, together with the modified copy used for the admissible-assay counts in Section 3.3.

**TABLE 3:** Run time and yield against target length, for truncations of the 1,026 bp hpt record, with template masking enabled and disabled. The record contains no repeated 15-mer, so the two halves of the table agree.

Length (bp)	Masks on: time (ms)	Candidates	Assays	Masks off: time (ms)	Candidates	Assays
300	62	85	1	60	85	1
500	96	147	3	92	147	3
1,000	240	317	167	248	317	167
1,026	246	323	167	248	323	167

## 3 Results

### 3.1 Behavior on a worked example

On the 391 bp example bundled with the tool, a design at default settings yields 111 candidates (23 F, 15 F1, 16 LF, 26 R, 16 B1, and 15 LB) and assembles them into five complete six-primer assays in approximately 90 ms. The first assay places F3 at 64–86, F2 at 92–111, LF at 116–138, F1 at 170–191, B1 at 200–221, LB at 228–247, B2 at 259–278, and B3 at 291–310, yielding a 187 bp F2-B2 fragment; FIP and BIP are both 48 nt. All eight components fall inside their intended Tm windows (outer 60.4 °C–61.6 °C, inner and loop 65.6 °C–66.9 °C), and all carry linguistic complexity of 78% or above (Figure 3; Table 4).

**FIGURE 3:**
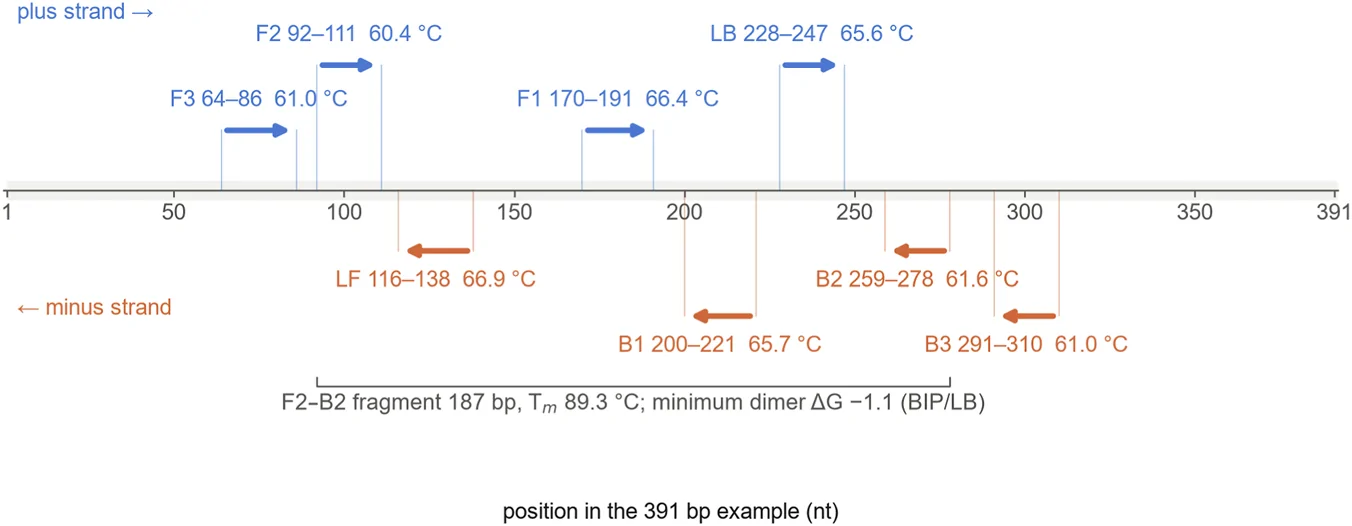
The first of the five complete assays returned for the bundled 391 bp example at default settings, drawn on the template. Plus-strand oligonucleotides are shown above the axis and minus-strand oligonucleotides below it, each with its coordinates and melting temperature. The bracket marks the F2-B2 fragment, with its length, melting temperature, and the minimum dimer free energy of the assembled assay at 65 °C. The full listing is given in Table 4.

**TABLE 4:** The first of the five complete assays returned for the bundled 391 bp example at default settings, exactly as reported by the tool. Positions are given on the plus strand; (+) and (−) denote the strand each oligonucleotide is read from. LC % and YR % are the linguistic-complexity values over the four-letter and the purine/pyrimidine alphabet.

Component	Sequence (5′–3′)	Length (nt)	T_m_ (°C)	GC (%)	LC (%)	YR (%)
F3 64–86 (+)	TCAGTTTACGAAAGGCAACCTAT	23	61.0	39.1	97	82
F2 92–111 (+)	CCAAGCAAGTAGGCATTGAG	20	60.4	50.0	81	65
LF 116–138 (−)	CGGGTCCGCGTTAAGCAATACAT	23	66.9	52.2	92	94
F1 170–191 (+)	CGCCCGACAGACGTTACATCAT	22	66.4	54.5	82	87
FIP = F1c + TTTTTT + F2	ATGATGTAACGTCTGTCGGGCGTTTTTTCCAAGCAAGTAGGCATTGAG	48	—	—	—	—
B1 200–221 (−)	AGGAAGCCAGCACCAAGTGTAT	22	65.7	50.0	82	86
LB 228–247 (+)	CCTTGACGGAACGCATCACC	20	65.6	60.0	89	96
B2 259–278 (−)	CGAAAGTACAGTACGGCGTT	20	61.6	50.0	78	67
B3 291–310 (−)	GATAGCCGAACGACTCACTT	20	61.0	50.0	86	85
BIP = B1c + TTTTTT + B2	ATACACTTGGTGCTGGCTTCCTTTTTTTCGAAAGTACAGTACGGCGTT	48	—	—	—	—
F2-B2 fragment	—	187	89.3	—	—	—
Minimum dimer ΔG at 65 °C	−1.1 (BIP/LB) kcal/mol	—	—	—	—	—

### 3.2 Throughput and scaling

Complete designs took 23–245 ms across the nine targets listed in Table 2, single-threaded. These are medians of seven runs; the run-to-run spread on one machine reached 15% of the median; therefore, Table 2 lists the minimum and maximum, and differences between settings smaller than approximately 20 ms should not be considered real. The run time follows the number of surviving candidates rather than the length of the target: the 1,066 bp tandem-repeat control, of which the masks remove 90.4%, leaves 24 candidates and is designed in 23 ms, while the 1,026 bp *hpt* record, which is not masked at all, leaves 323 and takes 245 ms. Truncations of the hpt record show that the cost increases with the candidate count as the window increases, from 62 ms at 300 bp to 246 ms at the full length, and masking makes no difference to any of them because that record contains no repeated 15-mer (Table 3; Figure 1A). When masking is applied, it dominates the cost instead: on the tandem-repeat control, the same design takes 23 ms with the repeat mask on and 264 ms with it off because the mask removes candidates before the quadratic assembly stage, rather than after it. The practical ceiling is therefore set by the number of surviving candidates and not by sequence length: enabling overlapping positions on the 391 bp example increases the candidate count from 111 to 353 and the run time from approximately 100 ms to 0.85 s, most of the increase being the thermodynamic screen of the 12,222 admissible assays that result (run-to-run variation between benchmark series is approximately 10 ms).

### 3.3 Yield and parameter sensitivity

The default parameter set is strict, and this is its most important practical property. Of nine targets designed at defaults, four returned no complete assay at all: the PrimerExplorer target discussed in Section 3.5 (419 bp), the NOS promoter (291 bp), the P-FMV promoter (554 bp), and the tandem-repeat control (1,066 bp); a fifth, the bla region, returned two. Candidates were abundant in three of the four failures (32, 64, and 88, respectively) and scarce only in the tandem-repeat control (24), where returning nothing is the correct outcome anyway, so for the natural targets, the constraint is not candidate supply but the combination of spacing, amplicon-size, and cross-dimer requirements imposed on assembly. The two parameters that dominate yield are the maximum F2-B2 distance and Tm window, but not in the same order for every target. On the worked example, decreasing the maximum F2-B2 distance from the default 200 bp to 150 bp gives no assays at all, while increasing it to 300 bp gives 15; widening the Tm window from 60 °C–62 °C to 58 °C–64 °C increases the yield from 5 assays to 54 (Figures 1B,C); the fact that the narrower 60 °C–61 °C window returns eight, more than the default, is a first sign of the order dependence discussed below. Disabling loop-primer design increases it from 5 to 41 because a four-primer assay has two fewer components to place and seven fewer cross-dimer tests to pass. Repeating the sweep on all nine targets (Supplementary Table S1) shows that the amplicon limit dominates only where assays are already being returned: on the four targets that yield nothing at defaults, widening it to 350 bp rescues none of them, whereas widening the Tm window rescues two. Table 5 gives the first single relaxation that returns a complete assay for each of those four. The NOS promoter needs 57 °C–65 °C and then returns two assays, and the P-FMV promoter needs 58 °C–64 °C and returns two; the PrimerExplorer target yields nothing under any single relaxation and needs the combination in Section 3.5; the tandem-repeat control yields nothing under any of the 10 relaxations tried, which is the correct outcome for it. A user who obtains no result should therefore widen the Tm window first and the amplicon limit second. The failure mode is still a silent empty table, and the tool would be improved by reporting which constraint was limiting. The reported assay count should be read as a lower bound on the design space because of the greedy thinning. With the thinning rule disabled for measurement, the worked example has 10 admissible dimer-free assays at default settings, of which five are reported; with overlapping positions allowed, it has 12,222, of which 2,540 are reported. The rule does what it is meant to, which is to keep the output readable, but the number it produces is not a count of possible assays, and small changes in candidate composition can change it substantially: widening the window to 60 °C–61 °C returns eight assays where the default 60 °C–62 °C returns five, although the narrower window admits fewer candidates. Nor does the rule remove near-identical assays. Applying to the tool’s own output the same criterion used against PrimerExplorer in Section 3.5, the five assays reported for the worked example fall into three groups, three of them differing only in B3. A diversity rule, rather than a monotonicity rule, would serve the user better and is the clearest improvement the assembly stage still needs. The gap is not confined to the worked example: with the thinning disabled, the five targets that return anything have 10, 70, 82, 15, and 360 admissible assays against 5, 19, 22, 2, and 167 reported (Table 6), so the reported number is between a seventh and half of the design space and must be read as a subset chosen by enumeration order rather than as a count. To see what a diversity rule would buy, the admissible assays of each target were clustered by the criterion used against PrimerExplorer in Section 3.5: two assays joined when they share four or more of their six core components, and three selections of the same size are compared. Taking the first n in enumeration order covers 2, 2, 2, 1, and 5 groups; the monotonicity rule the tool uses covers 3, 5, 5, 2, and 7; selecting greedily for maximum dissimilarity covers 3, 7, 8, 2, and 7. The present rule is therefore already much better than enumeration order and still short of what diversity-first selection would give on the two nptII targets. It has not been adopted in the shipped tool because the greedy maximum-dissimilarity pass is quadratic in the admissible set, which reaches 12,222 assays on the worked example once overlapping positions are allowed, and would dominate the run time in exactly the case where the output is least readable.

**TABLE 5:** The first single relaxation that returns a complete assay, for each of the four targets that return none at default settings. Relaxations were applied one at a time in the order: amplicon limit to 250, 300, and 350 bp; Tm window to 59–63, 58–64, and 57 °C–65 °C; loop primers off; then combinations. Widening the amplicon limit alone rescues none of the four.

Target	First relaxation that returns an assay	Assays	Admissible
PrimerExplorer V5 target	Tm 55 °C–66 °C, 350 bp, complexity 50%, 17–28 nt, loop primers off	82	217
NOS promoter	Tm window 57 °C–65 °C	2	9
P-FMV promoter	Tm window 58 °C–64 °C	2	2
Tandem-repeat control	None of the 10 relaxations tried	0	0

**TABLE 6:** Contribution of each filter, for every target. Each cell gives the number of assays reported and, after the solidus, the number admissible with the greedy thinning disabled. The masks alter nothing on any target because none of these records carries a repeated 15-mer; the 3′-end trinucleotide pattern is the filter that costs the most yield; and switching the dimer screen off changes no count anywhere, so at default settings, the screen contributes the value it reports rather than the assays it removes.

Target	Default	Both masks off	Complexity filter off	3′-end pattern off	Dimer screen off
Bundled example	5/10	5/10	5/10	5/15	5/10
PrimerExplorer V5 target	0/0	0/0	0/0	0/0	0/0
NOS promoter	0/0	0/0	0/0	0/0	0/0
nptII (neomycin)	19/70	19/70	39/110	71/216	19/70
nptII (kanamycin)	22/82	22/82	36/124	71/216	22/82
bla (ampicillin)	2/15	2/15	2/16	27/63	2/15
P-FMV promoter	0/0	0/0	0/0	0/0	0/0
hpt (hygromycin)	167/360	167/360	167/366	232/713	167/360
Tandem-repeat control	0/0	0/0	0/0	0/0	0/0

### 3.4 Repeat masking

The repeat control, a 1,066 bp sequence in which 21 copies of a 44 bp unit cover 86.7% of the length and the mask covers 90.4%, demonstrates the masking behavior directly. With repeat masking on, the tool proposes 24 candidates; with it off, it proposes 310, a 13-fold difference; the additional 286 candidates are placed on the tandem array itself. No complete assay is returned in either case, which is the correct outcome for a target of this composition. Across the remaining benchmark, the masks are close to inert. The repeat mask covers 9.1% of the PrimerExplorer target, whose submitted 419 bp carries a genuine internal duplication, and none of the six marker and promoter records, whose 15-mers are all unique within the sequence submitted; the low-complexity mask covers 7.4% of the P-FMV promoter and nothing else. Masking therefore changes a design only when the submitted record repeats itself, and this benchmark contains one such sequence by construction and one by accident. This measures what the filters remove, not what they prevent: no experiment in this study compares assays designed with the filters on and off, and the assays validated in Section 3.7 predate them. The masking and complexity filters rest on the mechanism of primer-derived background rather than on a demonstration that they reduce it.

### 3.5 Comparison with an archived PrimerExplorer V5 design

An archived PrimerExplorer V5 design (DesignId 170405125923) on a 419-bp target was available for direct comparison. PrimerExplorer returned 50 candidate sets spanning F2-B2 distances of 134–181 bp. These sets are highly redundant: although all 50 FIP sequences are distinct, they contain only 19 distinct F3, 9 distinct B3, and 8 distinct BIP sequences; thirteen sets share one identical B3/B2/B1c triple; and clustering sets that share four or more of their six primers collapses all 50 to 14 distinct groups. Therefore, the number of genuinely independent designs offered is closer to 14 than to 50. On the identical target, the tool described here returns no complete assay at default settings and none at moderately relaxed settings either; only at 55 °C–66 °C, 17–28 nt, 50% complexity, a 350 bp amplicon limit, and with loop primers disabled does it return 82 four-primer assays (Table 7). This is a stringency difference, rather than a defect on either side, and it should be stated plainly. PrimerExplorer applies no complexity or repeat filter and computes Tm at 4 mM Mg^2+^, rather than at 1 mM. Its nominally wider F3-F2 gap of 0–60 nt does not separate the two results here: across all 50 archived sets, that gap is 0–19 nt, so every one of them would satisfy this tool’s 0–20 nt rule. In one dimension, PrimerExplorer is stricter than the other, fixing F2-B2 at 120-160 nt, whereas the design reported here required a 350 bp limit before any assay was returned. All 50 archived sets are four-primer sets without loop primers, so the comparable rows in Table 7 are those with loop-primer design off. The archived design was evidently made with relaxed Tm settings: its F3 primers sit at 55.1 °C–57.2 °C, and its F1c segments at 60.2 °C–62.2 °C, below PrimerExplorer’s own default targets of 59 °C–61 °C for the outer primers and 64 °C–66 °C for F1c/B1c, which are close to the 60–62 and 65 °C–67 °C windows used here. The two tools therefore target the same Tm architecture, offset by the 1 °C–2 °C that their different salt conditions produce. The tool presented here is the more conservative of the two by design; a user who wants PrimerExplorer-like recall must ask for it explicitly. No run of the NEB tool, GLAPD, or LAVA was performed; therefore, the yield comparison above is limited to one tool on one target. Table 8 compares five freely available tools based on their documented features rather than yield. Two points should be noted: both the NEB LAMP Primer Design Tool and PrimerExplorer V5 design loop primers, and the NEB tool is NEB’s own implementation rather than a PrimerExplorer front end. The comparison can also be turned around: rather than asking what this tool returns on PrimerExplorer’s target, one can ask what its filters would do to PrimerExplorer’s primers. Every component oligonucleotide of the 50 archived sets (300 in all) and of the four validated assays in Section 3.7 (24 more) was put through the candidate filters, with the melting-temperature implementation checked first against the tool’s own output, which it reproduces to 0.01 °C. The linguistic-complexity threshold admits 85% of the archived oligonucleotides and 96% of the validated oligonucleotides, the 3′-end pattern admits 79% and 88%, and the self-complementarity test admits 94% and 92%, respectively; the default 60 °C-62 °C window admits none of the 300 and 13% of the 24. No archived set and none of the validated sets would therefore be proposed at default settings (Supplementary Table S2). That is a statement about conventions as much as about primers: PrimerExplorer designs at 50 mM Na^+^ and 4 mM Mg^2+^ to a wider window, and its F3 primers sit at 55.1 °C–57.2 °C on its own scale, so recomputing them at this tool’s fixed 55 mM, 1 mM, 0.2 µM condition moves them outside a 2 °C window almost by construction. It does, however, put a number on how strict the defaults are: they exclude the entire published comparison set, and a user who wants primers resembling published ones has to widen the window before anything else. The genuine distinctions lie in the client-side execution model, repeat and low-complexity masking of the template, linguistic-complexity filter, screening against user-supplied existing oligonucleotides, unrestricted multi-record input, and bisulphite mode.

**TABLE 7:** Design on the 419 bp target of PrimerExplorer V5, which returned 50 candidate sets (14 distinct groups) for the same input.

Settings	Candidates	Complete assays
Default (18–23 nt, 60 °C–62 °C, LC ≥ 70%, ≤200 bp, loops on)	32	0
Loop primers off	28	0
Max F2-B2 350 bp	32	0
Tm 58 °C–64 °C	49	0
55 °C–66 °C, 17–28 nt, LC ≥ 50%, ≤350 bp, loops off	71	82
55 °C–66 °C, 17–28 nt, LC ≥ 50%, ≤350 bp, loops on	96	0

**TABLE 8:** Feature comparison of five freely available LAMP primer design tools. The two right-hand columns record only what their documentation states; “not documented” means that the feature is not described, not that it is absent.

Feature	This tool	PrimerExplorer V5	NEB LAMP primer design tool	ConductScience LAMP primer designer	primd
Execution	Client-side, in the browser	Server-side	Server-side	Client-side, in the browser	Library, browser or Node.js
Target sequence leaves the user’s machine	No	Yes	Yes	No	No
Loop primer design	Yes	Yes	Yes	Yes	Yes, by default
Input	Plain, FASTA, multi-FASTA, tab-delimited, and NCBI accession	Plain, FASTA, GenBank, and multiple alignment	Plain, FASTA, and GenBank	Not documented	Sequence string passed by the caller
Input size limit	No hard limit (practical ceiling a few kb of unmasked template)	Not stated	100–2,000 nt analyzed window (longer records accepted with start/end coordinates)	Not documented	Not documented
Multi-record batch design	Yes	No	No	Not documented	A library, so batching is left to the caller
Template repeat masking	Yes (15-mer self-comparison)	No	No	Not documented	Not documented
Low-complexity masking	Yes	No	No	Not documented	Not documented
Linguistic complexity filter	Yes	No	No	Not documented	Not documented
Screen against user’s existing oligonucleotides	Yes	No (its fixed-primer function pins a primer to a region of the target)	No (as PrimerExplorer)	Not documented	Not documented
Bisulphite-converted templates	Yes	No	No	Not documented	Not documented
Dimer criterion	Combinatorial pre-filter, then nearest-neighbor ΔG of the assembled assay at 65 °C (default limit −6 kcal/mol; reported per assay)	Dimer ΔG threshold	Dimer ΔG threshold (default −2.5 kcal/mol; −3.5 for loop primers)	Not documented	Hairpin, self-dimer and hetero-dimer free energy at a settable annealing temperature
3′-end stability	3′-Trinucleotide pattern	ΔG ≤ −4 kcal/mol over the terminal bases	ΔG ≤ −4 kcal/mol over the terminal bases	Not documented	Not documented
Tm conditions	55 mM Mon, 1 mM Mg^2+^, 0.2 µM	50 mM Na^+^, 4 mM Mg^2+^, 0.1 µM	50 mM Na^+^, 8 mM Mg^2+^	Not documented	Not documented
F2-B2 constraint	≤200 bp (100–350 adjustable)	120–160 nt	120–160 nt	Not documented	Not documented
F2-F3 constraint	0–20 nt	0–60 nt	0–60 nt (15–60 recommended, optimum 40–60)	Not documented	Not documented
Registration required	No	No	No	No	Not applicable

### 3.6 Thermodynamic dimer screen

The combinatorial pre-filter decides only whether two oligonucleotides share a complementary stretch of six bases at the 3′end or eight bases internally; it says nothing about how stable the resulting duplex would be at 65 °C, and it never sees FIP and BIP as the 40–50 nt molecules that are synthesized. The thermodynamic screen described in Section 2.4 was calibrated against three types of assays whose fates are known (Table 9). All 215 assays reported on the nine benchmark targets at default settings have a minimum dimer ΔG between −3.7 and +4.6 kcal/mol, 38 of them below −3 kcal/mol, and none below −6; under permissive settings, the tail extends to −6.0 kcal/mol, and 4,105 of the 12,222 admissible assays of the overlapping-positions run lie below −3 kcal/mol. The 50 archived PrimerExplorer sets, which that tool accepted under its own free-energy criterion, spanned −4.0 to +10.4 kcal/mol on this scale. The four experimentally validated assays in Section 3.7 scored +0.8, +0.3, −2.4, and −4.5 kcal/mol; the last was a 36-bp self-alignment of the FIP of set B, the assay that gave the strongest ladder in Figure 4A. Therefore, a limit of −3 kcal/mol would have rejected a proven assay. However, this calibration compared unlike molecules, and correcting it changed the default. The four validated assays were synthesized with F1c joined directly to F2 as LAMP inner primers usually are, whereas this tool joins them through a TTTTTT linker and screens the molecule it would actually synthesize. Scoring the same sets both ways (Supplementary Table S3) shows that the linker is not innocent: it shifts the reported minimum by −1.2 kcal/mol on average and by between −9.4 and +6.5 kcal/mol in individual sets because a poly T tract is an additional pairing partner that the linker-free molecule does not carry. The *hpt* assay scores −2.4 kcal/mol as it was synthesized and −5.6 kcal/mol as this tool would build it, so a limit of −5 kcal/mol would reject the tool’s own construction of a functional assay. Recalibrating on like molecules, with every set scored with the linker the tool inserts, the 50 archived PrimerExplorer sets span −3.7 to +11.7 kcal/mol, and the validated assays span −5.6 to −0.3 kcal/mol, so −5 kcal/mol is the smallest limit that keeps every assay known to be functional. This is now the default, and it changes no yield reported here because the screen rejects nothing at default settings on any of the nine targets. The values printed using this tool are not comparable to the dimer free energies quoted elsewhere for linker-free primers. Table 10 shows how the limit affects the worked example. At default settings, all five assays pass at every limit tested; therefore, the screen does not change anything that a user sees except the reported value. Under a widened Tm window, it removes four of 58 assays at −5 kcal/mol and 22 at −2 kcal/mol. The counts are not monotonic in the limit, for the reason given in Section 3.3: rejecting an assay changes the reference against which the greedy thinning judges the assays that follow; thus, a stricter limit can allow more assays to pass. The reported minimum ΔG is the more useful product of the screen because it is the first quantity in the output that ranks the assays: the worked example’s five assays, indistinguishable by every other criterion, span −1.6 to −0.6 kcal/mol. The fact that the screen does not change anything at defaults is a general result and not a property of the example: across all nine targets, switching the screen off leaves the reported and admissible counts unchanged (Table 6). Its value at default settings is the number it reports, not the assays it rejects. The screen rejects what it is meant to reject. Four negative controls were built from the hpt assay by introducing a deliberate duplex: (i) the reverse complement of F3 appended to B3; (ii) B3 replaced by the reverse complement of F3; (iii) F1c replaced by the reverse complement of B1c; and (iv) a self-complementary 14-mer appended to F3. All four score below -6 kcal/mol, with the self-complementary construct reaching -15.0 kcal/mol, so all are rejected at the default limit. A milder control, 12 bases of the reverse complement of F2 appended to B2, scores −2.4 kcal/mol and passes at −6 but fails at −2 (Supplementary Table S4).

**TABLE 9:** Minimum dimer ΔG (kcal/mol at 65 °C, 349 mM sodium equivalent) of assays whose fate is known. “Admissible” assays are those the combinatorial rule accepts with the greedy thinning disabled; “reported” assays are the tool’s output at the stated settings with the screen off. The rows are of three different kinds and should not be read as one population: the worked example and the reported-assay rows are this tool’s own output and their outcome is unknown; the archived PrimerExplorer sets were accepted by that tool under its own criterion, and their outcome is likewise unknown; only the last row contains assays whose behavior at the bench has been observed.

Set	n	Minimum	Median	Maximum	Below −3	Below −5
Worked example, reported (default)	5	−1.6	−0.6	−0.6	0	0
Worked example, admissible (default)	10	−2.2	−0.6	−0.6	0	0
Worked example, admissible (Tm 58 °C–64 °C)	165	−6.0	−1.4	+1.9	21	6
Worked example, admissible (overlapping)	12,222	−3.8	−2.1	+3.0	4,105	0
Reported assays, all targets (default)	215	−3.7	−2.0	+4.6	38	0
PrimerExplorer V5, 50 archived sets	50	−4.0	+2.2	+10.4	1	0
Validated assays (Table 11): *hpt*/*npt*II/set A/set B	4	−4.5	-	+0.8	1	0

**FIGURE 4:**
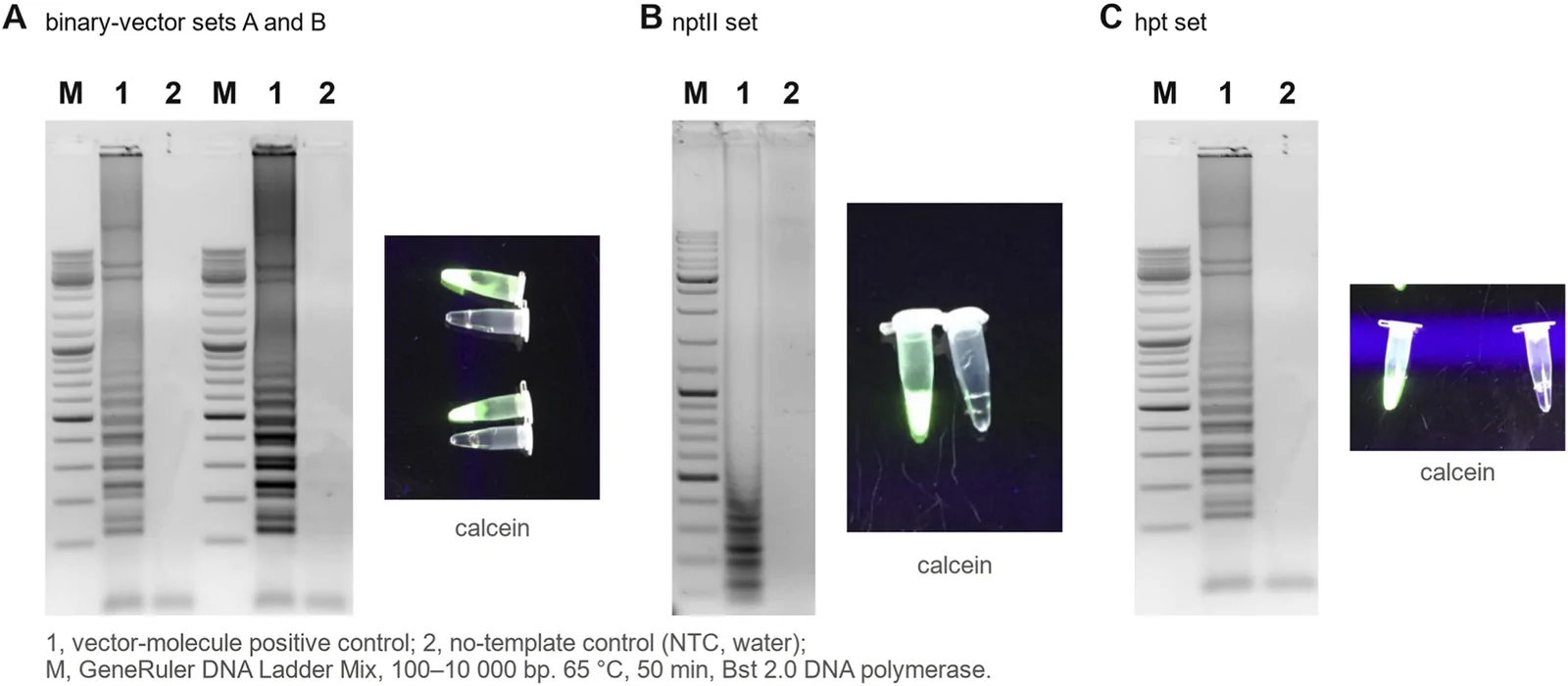
Experimental validation of four LAMP assays for transgene screening. Agarose electrophoresis (left of each panel) and calcein fluorescence of the reaction tubes (right) for **(A)** the two binary-vector sets A (oligonucleotides 5049–5052, left pair of lanes) and B (5053–5056, right pair), **(B)** the *nptII* set (5045–5048), and **(C)** the *hpt* set (5041–5044). All four sets give the characteristic LAMP ladder and bright calcein fluorescence from the positive control; the negative read-out is the absence of the ladder and of fluorescence rather than the absence of all signal, with the low-molecular-weight band in some negative lanes being unincorporated primer. Lanes are marked on each panel: 1, vector-molecule positive control; 2, no-template control (NTC, water); M, GeneRuler DNA Ladder Mix, 100–10,000 bp, as the size reference. The negative control is a no-template control only: no non-target DNA was run, so these panels do not address specificity against a background of related sequence. The assays shown were designed with an earlier implementation, and the figure is not offered as validation of the version described here.

**TABLE 10:** Complete assays returned from the worked example as a function of the dimer ΔG limit.

Settings	Screen off	−2	−3	−4	−5	−6 (default)
Default	5	5	5	5	5	5
Tm 58 °C–64 °C	58	36	45	46	54	54
Loop primers off	41	39	41	41	41	41
Overlapping positions allowed	2,540	1,097	1,763	2,540	2,540	2,540

### 3.7 Application: LAMP assays for transgene screening

Four four-primer LAMP assays were designed, developed, and evaluated experimentally as part of a program on DNA-based detection of genetically modified crops. Two of the four sets target unambiguous and individually identifiable markers. The hygromycin B phosphotransferase (*hpt*) set gives a 151 bp F3-B3 product, and the neomycin phosphotransferase II (*nptII*) sets a 199 bp product; both amplicon sizes were reproduced here exactly by *in silico* matching of the primers against the corresponding reference sequences, as were the tabulated FIP-BIP product sizes once the F1c and B1c 5′ extensions were added (Table 11). The remaining two sets amplified overlapping windows of an approximately 190-bp segment shared by a large family of binary cloning vectors. The source guideline annotates that segment as the CaMV 35S promoter, but the primers share no sequence with the authentic CaMV genome, and the assignment should be treated as unconfirmed, pending a database search. The conditions given here are those of the original laboratory records, which differ in scale and in two concentrations from the 50 µL formulation later printed in the guideline: reactions were run in 25 µL with 1× isothermal amplification buffer supplemented to 6 mM Mg^2+^, 1.4 mM dNTP, 0.15 M betaine, 1.6 µM each FIP and BIP, 0.2 µM each F3 and B3, and 8 U *Bst* 2.0 DNA polymerase, at 65 °C for 50 min, followed by 80 °C for 10 min. The products were resolved on agarose and read in parallel using calcein fluorescence. All four sets produced a characteristic LAMP ladder from a vector-molecule positive control, together with bright calcein fluorescence, while the no-template controls (NTCs) produced no ladder and no calcein signal (Figure 4); the low-molecular-weight band visible in some negative lanes is unincorporated primer, and the negative read-out is the absence of the ladder and of fluorescence, rather than the absence of all signals. The four panels are a selection from a larger body of work and should be read as such. The same laboratory records also contain the optimization that preceded them; magnesium, betaine, inner-primer concentration, and incubation time varied across runs, as did a conventional end-point PCR with the F3/B3 primers of the two vector-region sets, further primer sets directed at other vector features, and runs on transgenic plant material and non-transgenic plant DNA. None of that is reproduced here: it addresses the development of the assays rather than the behavior of the design tool, and it belongs with a separate account of that work. What is shown is the subset that documents the four assays described above, and nothing in this section should be read as a characterization of the assays’ specificity or sensitivity.

**TABLE 11:** LAMP assays for transgene screening validated experimentally. Outer product is the F3-B3 amplicon; inner product is the F2-B2 span plus the F1c and B1c 5′extensions. Sizes for the *hpt* and *nptII* assays were reproduced here by *in silico* matching against their reference sequences; the two binary-vector assays were matched against the vector records in which the segment occurs, and their target annotation is unconfirmed (Section 3.7). The F2 segment of the set A inner primer carries a degenerate position (Y) as it was ordered. This tool never emits a degenerate position, so it would not have proposed that primer, and set A is excluded from the free-energy calculations of Table 9 because the nearest-neighbor model has no parameters for an ambiguous base.

Target	Component	Sequence (5′→3′)	Length (nt)	Product (bp)
hpt	F3	GATCCCCATGTGTATCACTGGCA	23	Outer 151
hpt	FIP (F1c + F2)	CTCATCGAGAGCCTGCGCGAC + GATGGACGACACCGTCAGTGC	42	Inner 138
hpt	B3	TCAGGACATTGTTGGAGCCGAA	22	
hpt	BIP (B1c + B2)	TCGATGAGCTGATGCTTTGGGC + CGTGCACGAGGTGCCGGACTTCG	45	
nptII	F3	CATGGGTCACGACGAGATCCT	21	Outer 199
nptII	FIP (F1c + F2)	ATCTGGACGAAGAGCATCAGGG + CTTGAGCCTGGCGAACAGTT	42	Inner 169
nptII	B3	CTGCATACGCTTGATCCGGCT	21	
nptII	BIP (B1c + B2)	ACCGGCTTCCATCCGAGTACGTG + CCACCAAGCGAAACATCGCA	43	
Vector region, set A	F3	CCCAGGGGCTAGTATCTACGAC	22	Outer 165
Vector region, set A	FIP (F1c + F2)	GGAGTTCCCTTCAGTGAGCG + CGAGCGGCGAACTAATAACGYT	42	Inner 158
Vector region, set A	B3	GCCGTGCTGGACCGGGTTGAA	21	
Vector region, set A	BIP (B1c + B2)	CCTTGAAGTTGAGTATTGGCCGTC + GCCCGTAACTTTCGGTAGAGC	45	
Vector region, set B	F3	ACACCGAGCGGCGAACTAAT	20	Outer 167
Vector region, set B	FIP (F1c + F2)	GGAATCTCACCCATGCGCG + CGCTCACTGAAGGGAACTCCGGTT	43	Inner 163
Vector region, set B	B3	GGGCAGCAAGTCGGTTACCC	20	
Vector region, set B	BIP (B1c + B2)	TATTGGCCGTCCGCTCTACCGAAAG + CGTGCTGGACCGGGTTGAAT	45	

## 4 Discussion

The tool designs LAMP primer sets in the browser without installation, registration, or transmission of the target and adds four things that the hosted services do not offer: masking of dispersed repeats and low-complexity sequence in the template before candidates are enumerated, a linguistic-sequence complexity filter on every candidate oligonucleotide, a screen against the oligonucleotides a laboratory already holds, and a thermodynamic screen of every assembled assay for the most stable dimer its six molecules can form at the reaction temperature. Multi-record input and a bisulphite mode are included.

The defaults are strict, and this is the practical result a user needs to know: four of the nine benchmark targets returned no complete assay at defaults, and the two parameters that recovered the yield were the width of the Tm window and the maximum F2-B2 distance, in that order. Design takes 25–234 ms per target on one core; therefore, the relaxation path costs nothing. The experimental validation covers four four-primer assays on plasmid templates with an endpoint read-out, without loop primers, replicates, or a detection limit, and the assays themselves were designed using an earlier version of the tool. Within these bounds, the tool is a fast, private, and unusually conservative way to obtain a LAMP primer set.

### 4.1 Scalability

Design cost is governed by the number of surviving candidates rather than by target length because template masking, where it applies at all, removes candidates before the quadratic assembly stage. Across the nine benchmark targets, a complete design took 23–245 ms on one core, and the cost tracks the candidate count rather than the length: the 1,066 bp tandem-repeat control leaves 24 candidates after masking and designs in 23 ms, while the 1,026 bp *hpt* record, which carries no repeated 15-mer, leaves 323 and takes 244 ms (Section 3.2; Table 3). Masking pays only when the submitted record repeats itself: on the tandem-repeat control, the same design takes 29 ms with the repeat mask on and 287 ms with it off, while on a record without internal repeats, it changes neither the candidate list nor the run time. The practical ceiling is therefore a few hundred surviving candidates rather than a fixed template length: beyond that, the assembly stage and the thermodynamic dimer screen applied to each admissible assay begin to dominate, and the region of interest should be submitted rather than the whole record. The design runs in a single thread and is not parallelized.

### 4.2 Limitations

Specificity is assessed only within the submitted sequence. There is no genome-wide search or BLAST step; therefore, a primer set that is clean against its own template may still have binding sites elsewhere in a complex genome. The returned primer set should be screened separately, for example, using an *in silico* PCR tool (Kalendar et al., 2024), before synthesis. Output is not ranked: assays are printed in the order found after greedy thinning; thus, the user is shown a subset of the admissible assays chosen by enumeration order rather than by quality, and the final choice is left entirely to the user. The combinatorial pre-filter sees FIP and BIP only as their component regions; the assembled molecules are screened only by the thermodynamic stage. Masking is computed from the submitted record rather than from the genome, so it can act only on repeats the record itself contains; a dispersed repeat whose other copies lie outside the pasted region is invisible to it. Across the eight natural targets, the effect is small as seven carry no repeated 15-mer at all, so masking is a safeguard that rarely engages rather than a routine determinant of what can be designed.

The bisulphite mode is implemented but not benchmarked here.

No assay designed by the version described here has been evaluated at the bench. The four assays in Section 3.7 were designed with an earlier implementation, and their validation is an end-point, plasmid-template proof of concept without loop primers, replicates, or a detection limit; it establishes that the design principles yield workable assays, not that this version does. A prospective test of six-primer sets returned by the present version, with replicates, no-template controls, and a dilution series, is the obvious next step and is not attempted here. The computational evidence offered instead is of a different kind and has its own limits: it shows what the filters remove, what they would have removed from primers that are known to work, and that the screen rejects sets built to contain a dimer, but no computation can show that a set that passes will amplify.

The tool designs standard LAMP primer sets and nothing else, which bounds what it can offer to the SNP-genotyping variants of LAMP. Probe-based methods such as RART-LAMP (Zhang et al., 2023), which discriminates alleles with a dual-labeled RNA-modified probe cleaved by RNase H II, need a probe designed against the variant position; this tool does not design probes, does not place a variant deliberately at a primer 3′ end, and does not model allele-specific mismatch penalties, so it cannot by itself produce a genotyping assay of that kind. Probe-based LAMP genotyping has recently been freed from one of the constraints it used to place on primer design: Chang et al. (2026) show that the intrinsic DNA breathing of LAMP amplicons lets a probe reach a variant outside a loop region, so the primer set no longer has to reserve a transiently single-stranded loop for the probe. What is then asked of the design is only that the amplicon span the variant, which the bracketed-region markup of Section 2.1 already provides, and that no member of the set pair with the probe, which the screen against user-supplied oligonucleotides covers. Even so, extending the candidate stage to place a variant at a defined position within a primer and to score the allele-specific mismatch would be the change needed to serve genotyping directly.

Timings were measured under Node.js; only the worked example was timed in a browser. An empty result carries no diagnostic: the tool does not report which constraint was limiting. Finally, the whole design runs in a single thread; the practical ceiling is a few kilobases of unmasked template, and templates larger than that should be submitted as the region of interest rather than whole.

## Data Availability

The tool analyzed in this study is publicly available at https://primerdigital.com/tools/lamp.html, free of charge and without registration or licensing conditions. It is implemented entirely in client-side JavaScript and delivered unminified; therefore, the complete algorithm can be inspected in the browser; all computation and data handling occur on the user’s own device, and the only network request the page can initiate is the optional retrieval of a sequence by NCBI accession, which transmits the accession and not the target. The source modules are published at https://github.com/rkalendar/PCRtools. The benchmark harness, nine target sequences, design modules, and complete candidate and assay tables underlying every number reported here are provided as Supplementary Material. The tool is written in JavaScript (ECMAScript 2020) and runs in any current browser on any operating system; there is no server component, no build step, and no installation. The design logic is contained in nine modules, lamp.js, dna.js, dimers.js, dimers4.js, DNAtm.js, tsdr-nn.js, LowComplexitySequence.js, readseq.js, and tools.js, released under the GNU General Public License v3.0. Project page: https://github.com/rkalendar/PCRtools.
