# Supplementary material for: Browser-based design of loop-mediated isothermal amplification (LAMP) primer sets with template repeat masking and bisulphite support
Source: Front Bioinform. 2026 Oct 26;6:1992214. doi: 10.3389/fbinf.2026.1992214 (PMC13745653; doi:10.3389/fbinf.2026.1992214)
Supplement: Supplementary file 1 [file Table1.docx]

**Supplementary Material**

Browser-based design of loop-mediated isothermal amplification (LAMP) primer sets with template masking and assay-level dimer screening

Ruslan Kalendar

These four tables were added at revision, in answer to the referees. Every number was produced by reviewer_bench.js, reviewer_bench2.js and reviewer_bench3.js, which are included in the data archive together with the complete per-oligonucleotide output that Table S2 summarises.

**Table S1a.** Complete assays returned as a function of the max F2-B2 (bp), on all nine targets, every other setting at its default.

| **Target** | **120** | **150** | **200** | **250** | **300** | **350** |
| --- | --- | --- | --- | --- | --- | --- |
| Bundled example | 0 | 0 | 5 | 10 | 15 | 15 |
| PrimerExplorer V5 target | 0 | 0 | 0 | 0 | 0 | 0 |
| NOS promoter | 0 | 0 | 0 | 0 | 0 | 0 |
| nptII (neomycin) | 0 | 2 | 19 | 55 | 103 | 255 |
| nptII (kanamycin) | 0 | 2 | 22 | 70 | 135 | 271 |
| bla (ampicillin) | 0 | 0 | 2 | 51 | 141 | 192 |
| P-FMV promoter | 0 | 0 | 0 | 0 | 0 | 0 |
| hpt (hygromycin) | 0 | 1 | 167 | 294 | 346 | 613 |
| Tandem-repeat control | 0 | 0 | 0 | 0 | 0 | 0 |

**Table S1b.** Complete assays returned as a function of the outer Tm window (C), on all nine targets, every other setting at its default.

| **Target** | **60-61** | **60-62** | **59-63** | **58-64** | **57-65** |
| --- | --- | --- | --- | --- | --- |
| Bundled example | 8 | 5 | 17 | 54 | 76 |
| PrimerExplorer V5 target | 0 | 0 | 0 | 0 | 0 |
| NOS promoter | 0 | 0 | 0 | 0 | 2 |
| nptII (neomycin) | 16 | 19 | 107 | 157 | 254 |
| nptII (kanamycin) | 13 | 22 | 104 | 151 | 226 |
| bla (ampicillin) | 6 | 2 | 40 | 63 | 207 |
| P-FMV promoter | 0 | 0 | 0 | 2 | 0 |
| hpt (hygromycin) | 46 | 167 | 307 | 341 | 566 |
| Tandem-repeat control | 0 | 0 | 0 | 0 | 0 |

**Table S1c.** Complete assays returned as a function of the minimum linguistic complexity (%), on all nine targets, every other setting at its default.

| **Target** | **50** | **60** | **70** | **80** |
| --- | --- | --- | --- | --- |
| Bundled example | 5 | 5 | 5 | 0 |
| PrimerExplorer V5 target | 0 | 0 | 0 | 0 |
| NOS promoter | 0 | 0 | 0 | 0 |
| nptII (neomycin) | 39 | 39 | 19 | 7 |
| nptII (kanamycin) | 36 | 36 | 22 | 10 |
| bla (ampicillin) | 2 | 2 | 2 | 1 |
| P-FMV promoter | 0 | 0 | 0 | 0 |
| hpt (hygromycin) | 167 | 167 | 167 | 35 |
| Tandem-repeat control | 0 | 0 | 0 | 0 |

**Table S2.** Pass rate of published primer sets through the candidate filter chain. Every component oligonucleotide of the 50 archived PrimerExplorer V5 sets and of the four experimentally validated assays, assessed with the tool’s own filters and constants. The melting-temperature implementation reproduces the tool’s reported values to 0.01 °C. The 3′-end pattern is applied only to the oligonucleotides whose 3′ end is free in the assay.

| **Source** | **n** | **Length** | **Tm window** | **Complexity** | **3′-end pattern** | **Self-complementarity** | **All filters** | **Sets passing entire** |
| --- | --- | --- | --- | --- | --- | --- | --- | --- |
| PrimerExplorer V5 archive | 300 | 71 % | 0 % | 85 % | 79 % | 94 % | 0 % | 0 of 50 |
| experimentally validated | 24 | 88 % | 13 % | 96 % | 88 % | 92 % | 8 % | 0 of 4 |

**Table S3.** Effect of the TTTTTT linker on the reported minimum dimer free energy. Published inner primers are synthesised with F1c joined directly to F2; this tool inserts a TTTTTT linker and screens the molecule it would synthesise. Free energies in kcal/mol at 65 °C.

| **Set** | **As synthesised** | **With the tool’s linker** | **Shift** |
| --- | --- | --- | --- |
| hpt | -2.4 | -5.6 | -3.1 |
| nptII | 0.8 | -0.3 | -1.1 |
| vector set B | -4.5 | -2.0 | 2.5 |
| PrimerExplorer V5 archive, 50 sets | median shift 0.0 | range -9.4 to 6.5 |  |

**Table S4.** Deliberately strong-dimer sets as negative controls, built from the experimentally validated hpt assay. Free energies in kcal/mol at 65 °C.

|  | **Construction** | **Minimum dimer ΔG** | **Combinatorial rule** | **Rejected at −5** | **Rejected at −4** | **Rejected at −3** | **Rejected at −2** |
| --- | --- | --- | --- | --- | --- | --- | --- |
| negative control | reverse complement of F3 appended to B3 | -2.4 | rejects | no | no | no | yes |
| negative control | reverse complement of F2 appended to B2 | -2.4 | rejects | no | no | no | yes |
| negative control | B3 replaced by the reverse complement of F3 | -2.4 | rejects | no | no | no | yes |
| negative control | F1c replaced by the reverse complement of B1c | -0.8 | rejects | no | no | no | no |
| negative control | self-complementary 14-mer appended to F3 | -15.0 | rejects | yes | yes | yes | yes |
| reference | the unmodified hpt set | -2.4 | rejects | no | no | no | no |
